# A Unique Presentation and Clinical Course of Poststreptococcal Glomerulonephritis Following Recent Influenza Infection: A Case Report

**DOI:** 10.7759/cureus.111297

**Published:** 2026-06-22

**Authors:** Mark Rizk, Katarzyna Madejczyk

**Affiliations:** 1 Medicine, Lake Erie College of Osteopathic Medicine, Bradenton, USA; 2 Pediatric Emergency Medicine, AdventHealth Florida, Daytona Beach, USA

**Keywords:** immune complex glomerulonephritis, orbital edema, pediatric emergency department (ped), post-streptococcal glomerulonephritis (psgn), strep. pyogenes infections

## Abstract

Poststreptococcal glomerulonephritis (PSGN) is a well-characterized immune-mediated disease that typically follows group A streptococcal infection of the pharynx or skin. We present the case of a seven-year-old male who developed PSGN following a recent influenza virus infection without a clear history of preceding streptococcal illness. The patient presented with nausea, vomiting, edema, and hypertension, with laboratory findings including microscopic hematuria, proteinuria, decreased complement C3 levels, and elevated anti-streptococcal antibodies. His clinical course was notable for persistent hypertension requiring escalation of care to the pediatric ICU (PICU). Supportive management with antihypertensives, diuretics, and fluid restriction resulted in clinical improvement. This report highlights an atypical presentation and clinical course of PSGN, emphasizing the importance of maintaining a high index of diagnostic suspicion even in the absence of classic antecedent streptococcal symptoms and in the setting of a recent viral infection.

## Introduction

Acute poststreptococcal glomerulonephritis (PSGN) is a classic example of a type III hypersensitivity reaction that occurs following infection with Streptococcus pyogenes (group A streptococcus (GAS)) and, less commonly, groups C or G streptococci [1]. It remains the most common cause of acute nephritis in children worldwide, with an estimated 470,000 new cases annually and an incidence ranging from 9.5 to 28.5 per 100,000 individuals [2]. Although the global burden remains significant, the incidence has declined in developed countries since the 1970s [2-4].

PSGN most commonly affects children between five and 12 years of age and adults older than 60 years, with a male predominance. The risk of developing PSGN following streptococcal infection is estimated at 5-10% after pharyngitis and up to 25% following skin infections [2]. While a preceding streptococcal illness is often identified retrospectively, some patients present without recognized symptoms of pharyngitis or impetigo, making diagnosis more challenging and potentially delaying recognition of the underlying etiology. PSGN has been found to predispose individuals to chronic kidney disease, especially in the elderly, who typically experience more severe disease [3-5].

The pathogenesis of PSGN is mediated by immune complex deposition within the glomeruli, triggered by nephritogenic strains of GAS. Key antigens implicated include nephritis-associated plasmin receptor (NAPlr) and streptococcal pyrogenic exotoxin B (SpeB), which promote glomerular inflammation through the activation of the alternative complement pathway [3]. Circulating immune complexes deposit along the glomerular basement membrane, leading to complement activation, antibody binding, and subsequent glomerular injury. Histopathologic findings classically include subepithelial “hump-shaped” deposits on electron microscopy and granular deposition of IgG and C3 along the glomerular capillary walls and mesangium on immunofluorescence [2].

Clinically, PSGN presents along a broad spectrum ranging from asymptomatic microscopic hematuria to a full nephritic syndrome characterized by gross hematuria, proteinuria, edema, and hypertension. Management is primarily supportive, focusing on control of fluid overload and hypertension. Prognosis is generally favorable in pediatric populations, with most patients achieving complete recovery within weeks, although rare long-term renal complications have been reported [6]. These include proteinuria, hematuria, hypertension, and renal failure persisting for several months or years after resolution of the primary illness [6].

Recognition of PSGN may be particularly difficult when patients lack a clear history of antecedent streptococcal infection or present with symptoms attributable to concurrent illness. We report a pediatric case of PSGN that developed in temporal association with a recent influenza infection and in the absence of clinically apparent streptococcal pharyngitis or impetigo, highlighting a diagnostic scenario that may obscure recognition of this otherwise well-described disease.

## Case presentation

**Figure 1:**
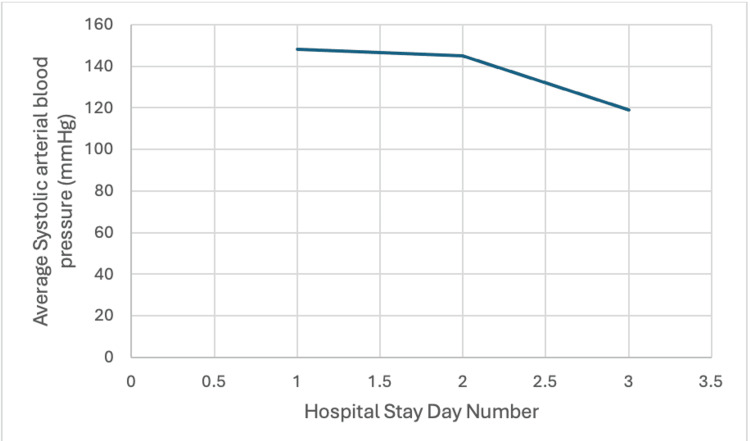
Timeline graph of the patient’s changing mean systolic arterial blood pressure over the three-day hospital course

A seven-year-old male with a past medical history significant for constipation presented to the pediatric emergency department with a four-day history of nausea, vomiting, facial rash, and bilateral hand swelling. On arrival, he was noted to be hypertensive with a blood pressure of 148/97 mmHg; all other vital signs were within normal limits (Figure 1).

The patient had experienced intermittent illness over the preceding 30 days following a confirmed infection with the influenza virus. On the day of presentation, his mother observed acute worsening of facial and hand swelling. He also reported mild, intermittent mid-abdominal pain during this period. There was no history of recent pharyngitis, skin infections, or other known streptococcal exposures. Physical examination revealed mild bilateral cheek swelling, dry mucous membranes, and nonpitting edema of the dorsum of both hands.

An abdominal radiograph demonstrated a nonobstructive bowel gas pattern with moderate stool burden, consistent with his history of constipation. Urinalysis was notable for nephritis (Table 1). Inflammatory markers, including C-reactive protein and erythrocyte sedimentation rate, were mildly elevated. Complete blood count demonstrated mild microcytosis with neutrophil predominance. Complement testing revealed decreased C3 levels with normal C4 (Table 1).

**Table 1:** Laboratory findings CBC: complete blood count; WBC: white blood cell; MCV: mean corpuscular volume; MCH: mean corpuscular hemoglobin; MCHC: mean corpuscular hemoglobin concentration; RDW: red cell distribution width; BMP: basic metabolic panel; CO_2_: carbon dioxide; BUN: blood urea nitrogen; UA: urinalysis; RBC: red blood cells; ESR: erythrocyte sedimentation rate; CRP: C-reactive protein

Lab findings		
	Result	Reference range
CBC		
WBC, x10^3^/µL	5.5	4.5-12.0
Neutrophils, %	65	40-60
Lymphocytes, %	30	25-45
Monocytes, %	3	3-9
Eosinophils, %	1	1-4
Basophils, %	0	0-1
Hemoglobin, g/dL	13.5	11.5-15.5
Hematocrit, %	38	35-45
Platelets, x10^3^/µL	220	150-450
MCV, fL	75	77-95
MCH, pg	28	25-33
MCHC, g/dL	34	32-36
RDW, %	12.1	11.4-13.5
BMP		
Sodium, mEq/L	139	135-145
Potassium, mEq/L	4	3.5-5.1
Chloride, mEq/L	105	102-112
CO_2_ (bicarbonate), mEq/L	23	20-28
BUN, mg/dL	25.6	7-20
Creatinine, mg/dL	0.76	0.26-0.61
Glucose (fasting), mg/dL	78	60-100
Calcium, mg/dL	8.9	8.5-10.2
UA		
Color	Yellow	Yellow/amber
Appearance	Clear	Clear
Specific gravity	1.021	1.003-1.035
pH	5.3	4.8-7.8
Protein	1+	Negative/trace
Glucose	Negative	Negative
Ketones	Negative	Negative
Blood	3+	Negative
Nitrites	Negative	Negative
Leukocyte esterase	Negative	Negative
WBC, /HPF	0	0-5
RBC, /HPF	>50	0-3
Complement		
C3, mg/dL	38	90-180
C4, mg/dL	22	10-40
Inflammatory markers		
ESR, mm/hr	67	3-13
CRP, mg/L	45	<3
Antibodies		
Anti-streptolysin O, IU/mL	430	<150
Anti-DNase B, U/mL	620	<300

**Figure 2:**
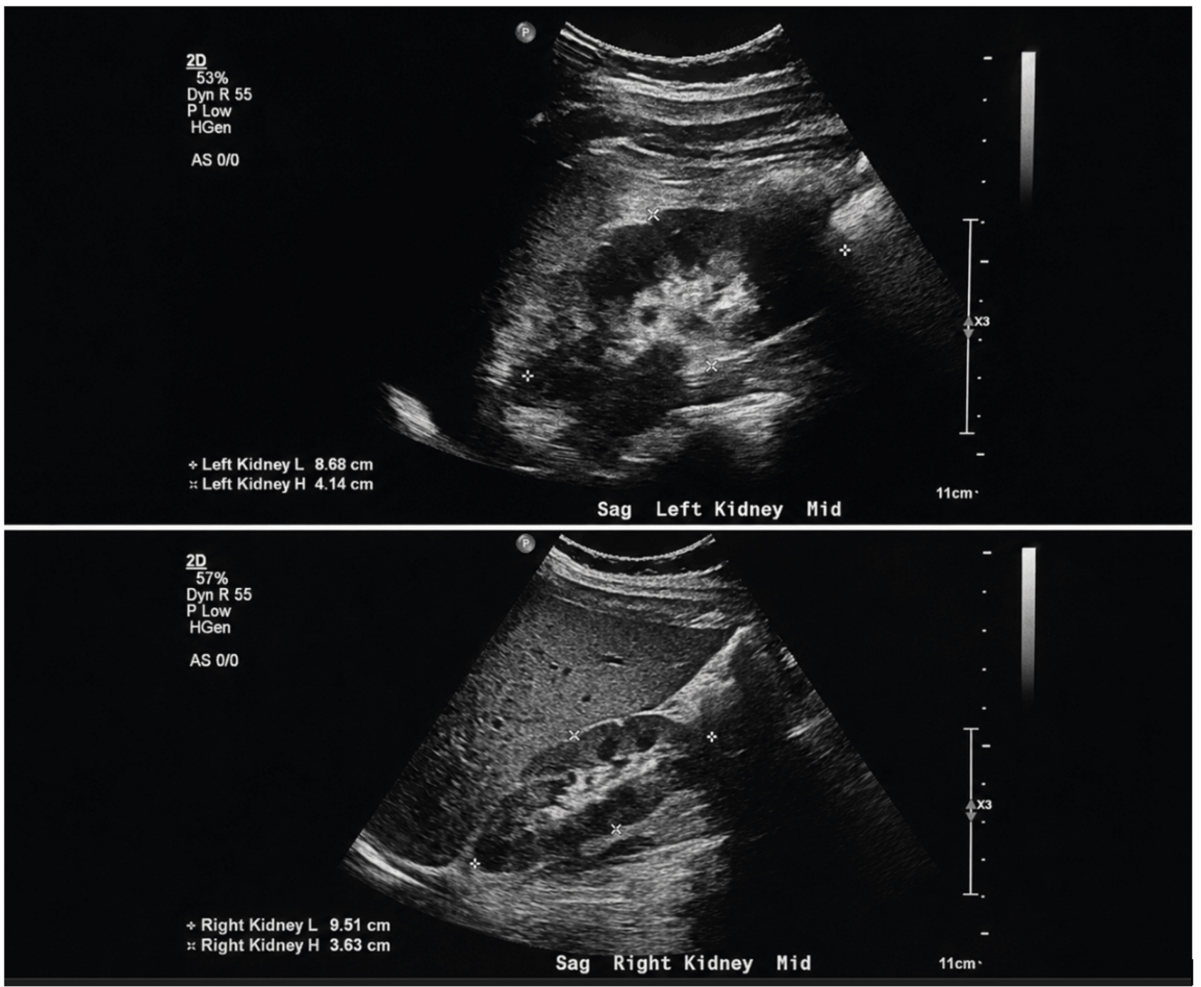
Left and right renal ultrasounds demonstrating normal bilateral kidney morphology

An electrocardiogram was obtained to assess for evidence of chronic hypertension and showed a normal sinus rhythm without evidence of left ventricular hypertrophy. Renal ultrasound demonstrated normal bilateral kidney morphology; however, an incidental small left-sided pleural effusion was identified (Figure 2).

**Figure 3:**
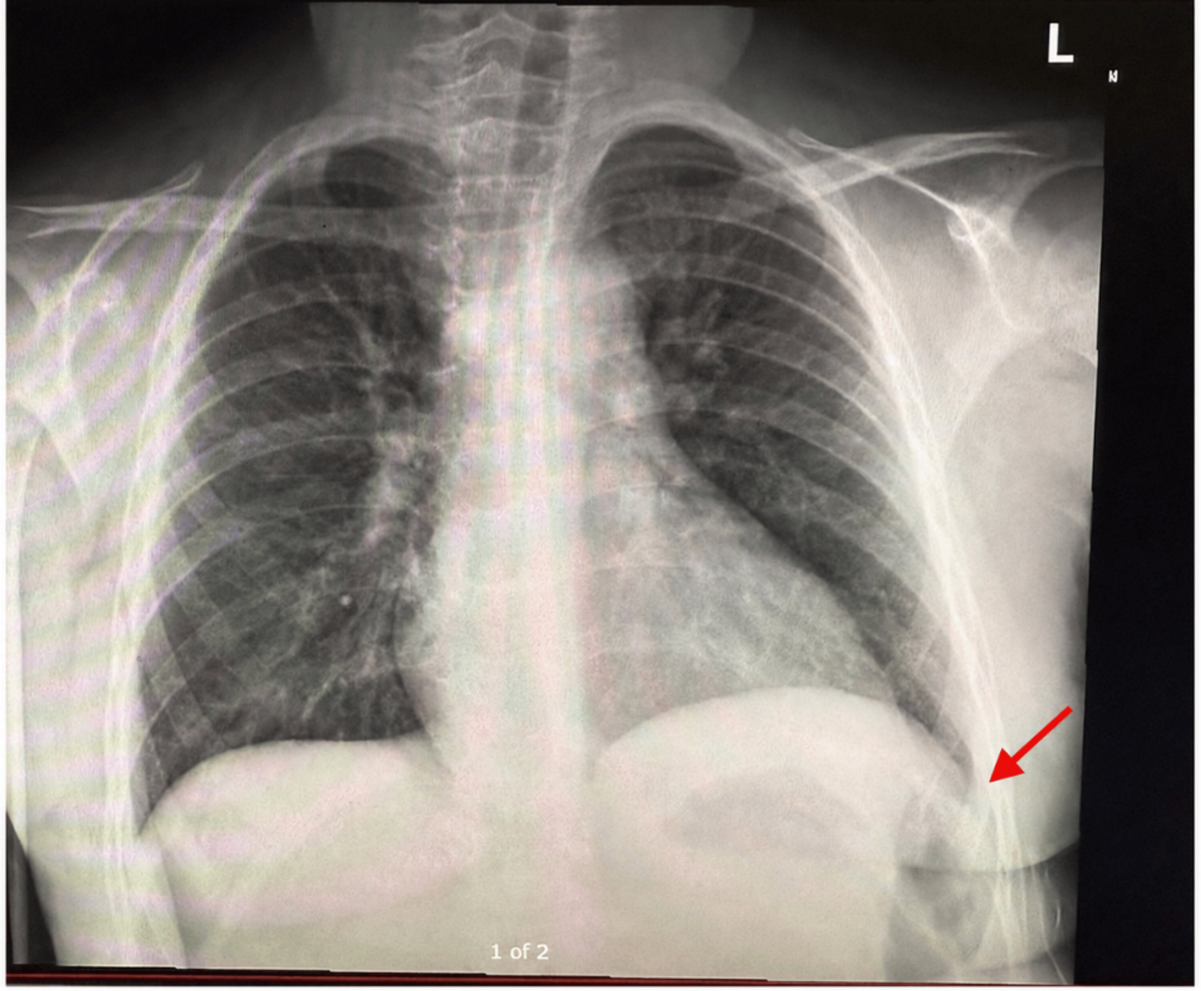
Chest X-ray confirming the incidental small left pleural effusion (red arrow) found on renal ultrasound

This was subsequently confirmed on chest radiography (Figure 3).

Group A streptococcal PCR testing returned positive, prompting consultation with pediatric nephrology. Serologic testing revealed elevated anti-streptolysin O and anti-DNase B titers, supporting the diagnosis of poststreptococcal glomerulonephritis (Table 1).

Despite initial management, the patient remained hypertensive but asymptomatic. Two doses of hydralazine were administered without significant improvement, necessitating transfer to the pediatric intensive care unit. Antihypertensive therapy with amlodipine and diuresis with furosemide were initiated, along with fluid restriction for volume management. By hospital day three, his blood pressure improved, with stabilization of renal function (BUN 29 mg/dL, creatinine 0.72 mg/dL) (Figure 1, Table 1). This case highlights an unusual clinical course of poststreptococcal glomerulonephritis following recent influenza virus infection, emphasizing the need to consider PSGN in pediatric patients with nephritic features despite the absence of classic preceding streptococcal symptoms.

## Discussion

PSGN remains one of the most common causes of acute nephritic syndrome in children worldwide and classically occurs following streptococcal pharyngitis or skin infection [1-2]. Most pediatric patients present with edema, hematuria, hypertension, and transient hypocomplementemia approximately one to three weeks after pharyngitis or up to six weeks after skin infection [2]. Our patient demonstrated several hallmark clinical findings of PSGN, including microscopic hematuria, proteinuria, edema, hypertension, elevated antistreptococcal antibodies, and low C3 levels with preserved C4, supporting the diagnosis of complement-mediated glomerular injury.

Despite these classic laboratory findings, the clinical presentation was atypical because the patient lacked a known recent streptococcal illness and instead had a prolonged symptomatic course following confirmed influenza infection. Similar atypical presentations have been increasingly recognized in the literature, particularly among pediatric patients with subclinical streptococcal infections [6]. Stoian et al. have reported that PSGN may present with variable clinical severity and occasionally without a clearly identifiable antecedent infection, complicating diagnosis and delaying recognition [6]. Our patient similarly lacked classic pharyngitis or impetigo symptoms despite positive streptococcal serologies and PCR testing.

Recent literature has also emphasized the complex immunologic mechanisms underlying PSGN. Dhakal et al. described how nephritogenic streptococcal antigens, including nephritis-associated plasmin receptor and SpeB, contribute to complement activation and glomerular inflammation through immune complex deposition [3]. These inflammatory pathways may potentially be amplified by preceding viral infections that dysregulate the host immune response. Influenza infection has been associated with transient immune dysfunction and increased susceptibility to secondary bacterial infections, which may explain the temporal relationship observed in this case. Although a direct causal relationship between influenza and PSGN has not been established, viral illnesses may obscure the identification of antecedent streptococcal infection or contribute to exaggerated immune activation. Therefore, as in our patient, the preceding severe influenza infection may have obscured or even coincided with a subclinical streptococcal infection. 

Our patient additionally demonstrated severe hypertension requiring escalation of care to the pediatric ICU (PICU). While most pediatric PSGN cases are self-limited and managed conservatively, severe hypertension and fluid overload remain important complications [2]. Pleural effusions and pulmonary edema have been reported in more severe presentations secondary to sodium and fluid retention from reduced glomerular filtration [1]. The incidental pleural effusion identified in this patient likely reflected early volume overload associated with acute nephritic syndrome. Prompt recognition and management with antihypertensive therapy, diuresis, and fluid restriction resulted in stabilization without progression to respiratory compromise or dialysis requirement.

Long-term prognosis in pediatric PSGN is generally favorable; however, persistent urinary abnormalities, hypertension, and chronic kidney disease have been documented in some patients [5-6]. Munif et al. have highlighted that persistent complement activation and inflammatory injury may contribute to long-term renal sequelae in susceptible individuals [5]. Consequently, continued outpatient follow-up remains important even in patients who demonstrate rapid clinical improvement during hospitalization.

This case contributes to the current literature by describing an unusual presentation of PSGN following recent influenza infection in the absence of classic streptococcal symptoms. It further highlights the importance of considering PSGN in pediatric patients presenting with edema, hypertension, and nephritic urinary findings despite an atypical infectious history.

## Conclusions

This case report illustrates an atypical presentation of PSGN occurring after a recent severe influenza virus infection without clear preceding streptococcal symptoms. It highlights the potential for viral illnesses to obscure the clinical timeline or contribute to immune-mediated renal disease. Clinicians should maintain a high index of suspicion for PSGN in pediatric patients presenting with nephritic features, regardless of reported prior impetigo or pharyngitis infections. Prompt diagnosis and supportive management are essential to optimize outcomes and prevent complications.

## References

[1] Rodriguez-Iturbe B, Haas M (2026). Rodriguez-Iturbe B, Haas M: post-Streptococcal glomerulonephritis. https://europepmc.org/article/nbk/nbk333429#impact.

[2] (2026). Poststreptococcal glomerulonephritis: clinical features, diagnosis, and management. http://com.lecom.idm.oclc.org/contents/poststreptococcal-glomerulonephritis-clinical-features-diagnosis-and-management.

[3] Dhakal AK, Shrestha D, Preston R, Lennon R (2025). Acute post-streptococcal glomerulonephritis in children-treatment standard. Nephrol Dial Transplant.

[4] Miller KM, Van Beneden C, McDonald M (2022). Standardization of epidemiological surveillance of acute poststreptococcal glomerulonephritis. Open Forum Infect Dis.

[5] Munif MR, Hart RA, Rafeek RA (2024). Mechanisms that potentially contribute to the development of post-streptococcal glomerulonephritis. Pathog Dis.

[6] Stoian M, Scarlat G, Dona B, Procopiescu B, Ciofu C (2022). Clinico-pathological correlations of poststreptococcal glomerulonephritis. Intern Med.

